# Incidence, severity and orthodontic treatment difficulty index of impacted canines in Saudi population

**DOI:** 10.4317/jced.54385

**Published:** 2018-04-01

**Authors:** Maged-Sultan Alhammadi, Hanan-Ahmed Asiri, Abeer-Abdulkarem Almashraqi

**Affiliations:** 1 Assistant professor, Department of Preventive Dental Sciences, Division of Orthodontics and Dentofacial Orthopedics, College of Dentistry, Jazan University, Jazan, Saudi Arabia; 2 Intern student, College of Dentistry, King Khaled University, Abha, Saudi Arabia; 3 Assistant professor, Department of Maxillofacial Surgery and Diagnostic Sciences, College of Dentistry, Jazan University, Jazan, Saudi Arabia

## Abstract

**Background:**

The objective of this study was to assess the incidence, severity and orthodontic treatment difficulty of impacted maxillary canines in Saudi population.

**Material and Methods:**

This retrospective study included an investigation of panoramic radiographs for patients attended College of Dentistry, Jazan University, Saudi Arabia. The incidence of canine impaction and orthodontic treatment difficulty index of maxillary canine impaction was assessed based on; (1) patient age, (2) vertical position, (3) buccolingual position, (4) horizontal position, (5) incisors alignment, (6) canine space, (7) midline coincidence, (8) rotation of impacted tooth. Statistical analyses were calculated by independent Chi-Square test. A P value of less than 0.05 was considered significant.

**Results:**

Canine impaction was found in (1.9%) of the population. Bilateral canine impaction was present in 22.3% of the patient with impacted canines. Ninety two percent had impacted maxillary canines only while 7.5% had impacted maxillary canines with other impacted teeth. The ratio of maxillary to mandibular impaction was about 10:1. Females (69.4%) had more impacted canines than males (30.6%) with no significant sex predilection. Orthodontic treatment difficulty index was statistically significant (*P* ≤0.05) in males more than females. Males revealed statistically significant (*P* ≤0.05) difficulty regarding canine angulation and the vertical position while females showed significant difficulty regarding dental midline and incisors irregularity or crowding of incisor segment.

**Conclusions:**

Prevalence of maxillary canine impaction in Jazan is within the range of impacted canine in other populations. Females showed more canine impactions than males while the orthodontic treatment difficulty index is more in males than females.

** Key words:**Incidence, Jazan, impaction, maxillary canines, difficulty index.

## Introduction

Eruption of teeth is a complex process, therefore early, delayed or even failure of teeth eruption may occur. Once the scheduled time of teeth eruption passed, these teeth considered as an impacted teeth (1). Impaction of teeth is one of the common dental abnormalities. Third molars are the most commonly impacted teeth, followed by permanent canines (2). The exact etiology of teeth impaction is unknown. Several etiological factors for canine impactions have been proposed: localized, systemic, or genetic factors. The most common localized factor is arch length-tooth size discrepancy. These factors are mainly for labilally impacted canines, Jacoby’s (3) found that only 17% of labially impacted canines had sufficient space, while 85% of palatally impacted canines had sufficient space.

Two main theories associated with displaced maxillary canines are the genetic theory and guidance theory (2). The genetic theory considers the genetic factors as a primary origin of palatally displaced maxillary canines. The guidance theory points that the canine erupts guided by the root of the lateral incisor, and if the root of the lateral incisor is malformed or absent, the canine will not erupt (4). The sequelae of canine impaction includes; malpositioning of the impacted tooth, migration of the neighboring teeth and loss of arch length, internal resorption, dentigerous cyst formation, external root resorption of neighboring teeth, and local infection of partially erupted canines (5).

There are several methods for diagnosis of impacted canines which includes; chronological age, clinical examination, and radiographic examination. Ericson and Kurol (6) stated that the absence of the “canine bulge” at earlier ages should not be considered as indicative of canine impaction. In their examination of 505 schoolchildren between 10 and 12 years of age, they found that 29%,5% and 3% of the children had non-palpable canines at 10, 11 and 12 years, respectively. Therefore, for an accurate diagnosis, the clinical examination should be supplemented with a radiographic evaluation. The diagnostic information obtained from panoramic radiography is valuable for the overview, prediction, and follow up of tooth eruption and treatment results. It would be advantageous to use panoramic radiographs in localizing impacted maxillary canines as it was the most commonly recommended screening radiograph, delivers less radiation dose, easy to perform, cost effective and readily available (7).

The management of impacted canines is important in terms of esthetics and function. The management of impacted canines usually involves different options; radiographic monitoring for cystic formation, interceptive treatment, surgical exposure and orthodontic traction and finally surgical extraction. Orthodontists must formulate treatment plans that are in the best interest of the patient and they must be knowledgeable about the treatment difficulty and variety of treatment options.

Prediction of treatment success has been based mainly on clinical personal experience. The presence of a system that offered an improved assessment of the likely difficulty of aligning impacted canine would be beneficial for both patient and clinician. Pitt and colleagues (8) developed treatment difficulty index for unerupted maxillary canines. This index based on age, angulation of midline, vertical position, buccolingual position, horizontal position, alignment of upper incisor, canine space within the dental arch, midline deviation and canine rotation.

Up to date, there is no study assessed the prevalence of impacted canines in Jazan, Saudi Arabia and the difficulty of orthodontic treatment was not considered in previous study based on epidemiological data. The purpose of this study was to report the prevalence of impacted canine teeth according to the age, gender and severity of impaction as well as the orthodontic treatment difficulty based on treatment difficulty index among Saudi population.

## Material and Methods

This was a population-based, retrospective cross sectional study based on the surveying of panoramic radiographs of patients attended the outpatient clinic, College of Dentistry, Jazan University, Saudi Arabia from January 2015 to October 2016. It was approved by the College Research Committee, College of Dentistry, Jazan University, Saudi Arabia. The inclusion criteria were: (1) chronological age range: 14 - 40 years, (2) all teeth are present with/without the third molars, (3) no interproximal caries or restoration, (4) no crown or bridge restoration. All panoramic radiographs were taken with the Orthophos XG 5 (Sirona Dental Systems, Bernsheim, Germany) and the magnification factor was 1:1. All reported measurements were adjusted according to this factor. Data regarding patient age, sex were obtained from patients’ main data in the R4 system. All panoramic radiographs were investigated by two observers in two time interval to assess intra- and inter-examiner reliability of measurements.

The severity score of maxillary canine impaction was assessed based on:

1. Angulation between the midsagittal plane and the long axis of the impacted tooth [mild (10-15°), moderate (15-30°) and severe (˃ 30°)].

2. Horizontal overlap [mild (no overlap), moderate (˂ half of the lateral incisor overlapped) and severe (˃ half of the lateral incisor overlapped)].

3. Apex position [mild (in the area of canine apex), moderate (in the area of the first premolar apex) and severe (in the area of the second premolar apex)].

4. Vertical overlap [mild (between the cementoenamel junction and the middle of the adjacent incisors), moderate (between the middle and the apices of the adjacent incisors) and severe (above the apices of the adjacent incisors)].

**Figure 1:**
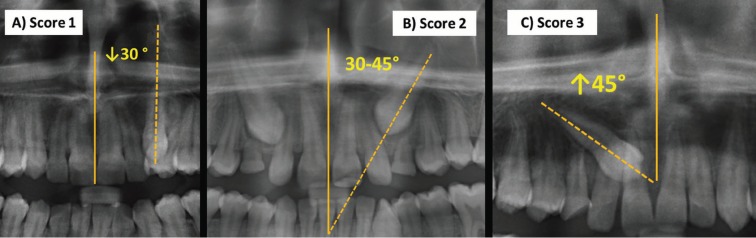
Angulation of the impacted canine to the midsagittal plane A) Score 1: less than 30 degree, B) Score 2: between 30–45 degree, C) Score 3: more than 45 degree.

**Figure 2:**
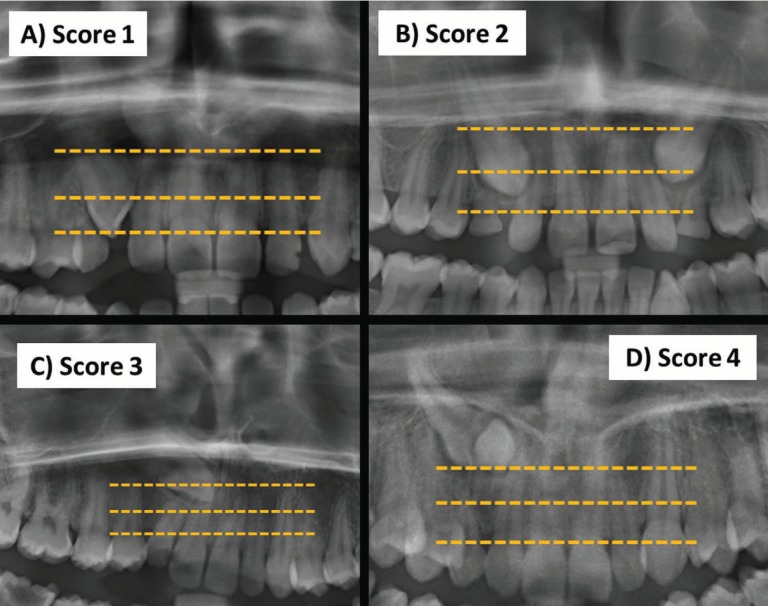
Vertical position of the impacted canine A) Score 1: canine cusp tip at the level of the cementoenamel junction of the adjacent incisor, B) Score 2: canine cusp tip at the middle of root the adjacent incisor, C) Score 3: canine cusp tip within the apical third of root the adjacent incisor, D) Score 4: Canine cusp tip above the apical third of root the adjacent incisor.

**Figure 3:**
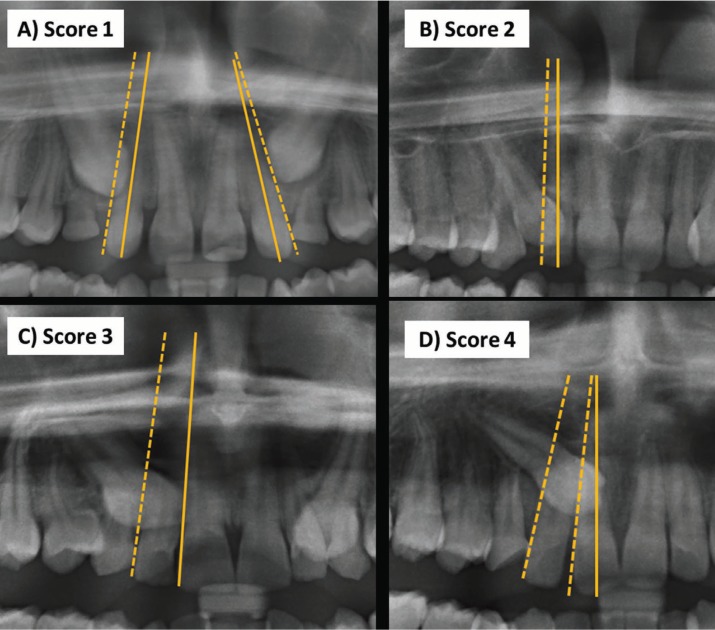
Horizontal position of the impacted canine A) Score 1: canine overlapping up to half the width of the lateral incisor, B) Score 2: canine overlapping over to half the width of the lateral incisor, C) Score 3: canine completely overlapping the lateral incisor, D) Score 4: canine overlapping up to half the width of the central incisor.

The treatment difficulty index for unerupted maxillary canines was scored using the index proposed by Pitt *et al.* (8). The following nine factors were considered; (1) patient age, (2) angulation to midline (Fig. 1), (3) vertical position (Fig. 2), (4) buccolingual position, (5) horizontal position (Fig. 3), (6) incisors alignment, (7) canine space, (8) midline coincidence, (9) rotation of impacted tooth. The grading system and weighting factor of each variable presented in  Table 1. The weighting of each factor in the difficulty score was set according to that proposed by Pitt *et al.* (8) ( Table 1).

**Table 1:**
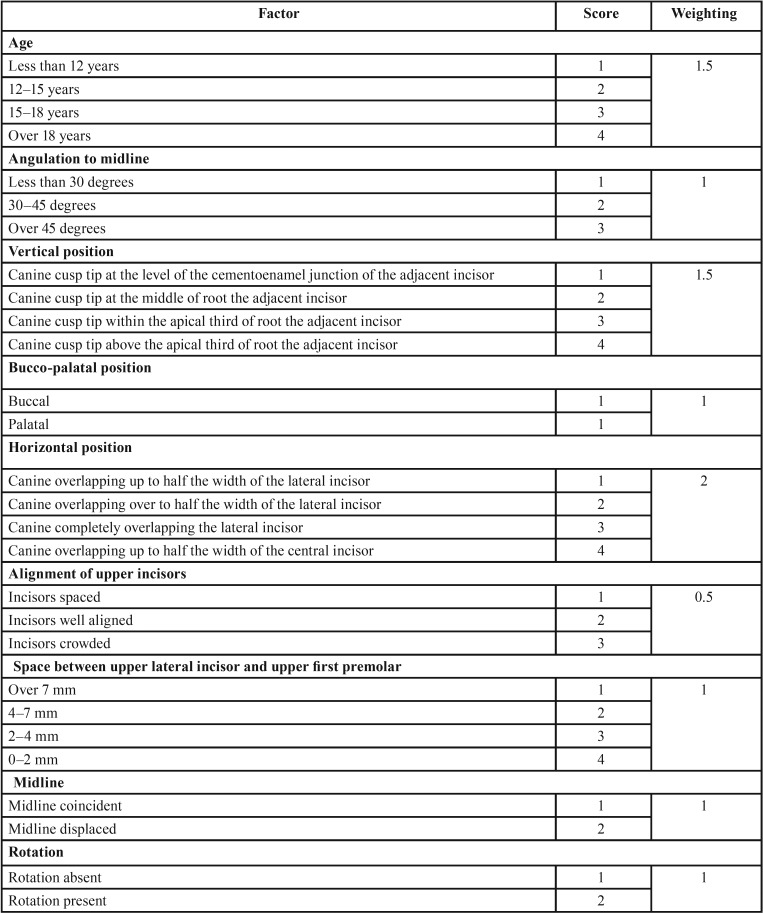
Grading system of the orthodontic treatment difficulty index.

-Statistical analysis

Data were handled and analyzed using IBM SPSS Statistics for Windows, Version 21 (Armonk, NY: IBM Corp.). For statistical descriptions, frequencies along with percentages and means with standard deviations were calculated and presented for all, and by gender. Associations of the severity scores of impacted upper canine with gender were analyzed by Chi-Square test. Gender differences in treatment difficulty index were analyzed by independent t-test. A *P* value of less than 0.05 was considered significant.

## Results

Seven thousands and fifty three panoramic radiograph were scanned for this study. Based on the selection criteria, only 937 patients’ panoramic radiographs fulfilled the inclusion criteria (465 males and 472 females) and were included in the final evaluation of impacted teeth. Maxillary canine impaction with or without other impactions occurred in (1.9%) of the scanned radiographs.

Bilateral canine impaction was present in 30 patients out of 134 patients which represent 22.3% of the patient with impacted canines ( Table 2). From the total evaluated sample, 92.5% had impacted maxillary canines only while 7.5% had impacted maxillary canines with other impacted teeth. The ratio of maxillary to mandibular impaction was about 10:1. There is no difference in prevalence between the right and left side in both arches. Based on the quadrant distribution, the descending order of impaction was in upper left (53%), upper right (47%), lower right (6.7%) and finally lower left quadrant (3%) ( Table 2). Regarding sex distribution, 30.6% of males and 69.4% of females had impacted maxillary canines with no significant sex predilection.

**Table 2:**
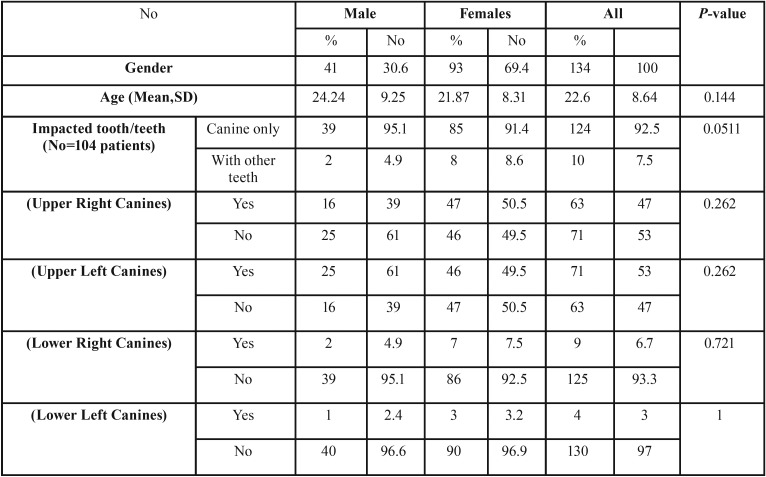
The frequencies, percentages and results of independent student t test for comparison between age, impacted canines in males and females.

The results showed very good intra-examiner and inter-examiner reliability (0.915 – 0.981 and 0.884 – 0.940, respectively). Assessment of the severity of impaction revealed that there were no statistical significant differences between both genders in all evaluation factors ( Table 3). Seventy seven percent of impacted maxillary canines had severe angulation relative to the midsagittal plane (> 30°). Most of impacted canines (57%) were severely overlapping more than half of the lateral incisor root. The root apexes of 47% of impacted canines were located in the first premolar apex position. The cusp tips of maxillary impacted canines were positioned between the cementoenamel junction (CEJ) and the middle of the adjacent incisors in 63.4% of the total impacted canines.

**Table 3:**
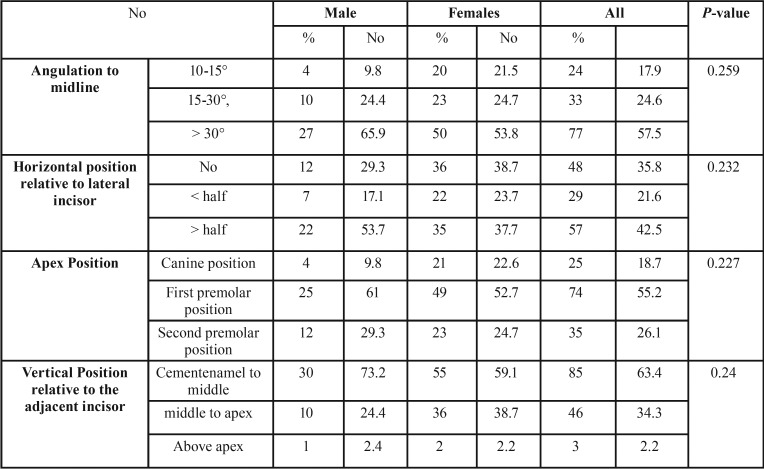
The frequencies, percentages and results of independent student t test for comparison of the parameters for maxillary canine impaction severity in males and females.

Regarding the difficulty of orthodontic treatment of analyzed impacted maxillary canines, there are statistical significance differences between males and females in the total difficulty index (*P*≤ 0.05) ( Table 4). When each factor was evaluated separately, four out of the nine studied variables were statistically significant between both genders. Those factors are in the significance order, angulation of the impacted canine to the midsagittal plane, dental midline coincidence with the skeletal midline, alignment of incisors teeth and finally the vertical position of impacted canine relative to the adjacent incisors.

**Table 4:**
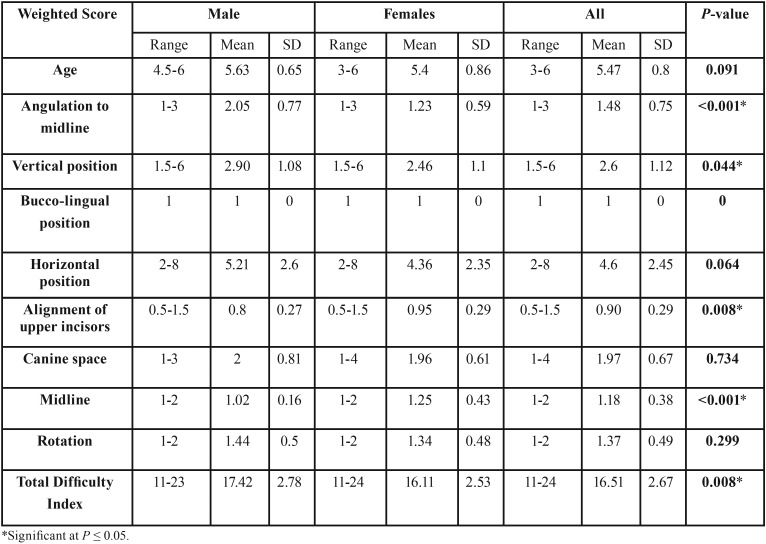
The Mean and standard deviation and results of independent student t test for comparison between all factors of difficulty index in males and females.

Males showed more difficulty regarding angulation of the impacted canine to the midsagittal plane as most of the canines have an angulation of 30–45 degree or more to the midsagittal plane (2.05±0.77). The same extend to the vertical position of impacted canine relative to the adjacent incisors as most of the cusp tips of impacted canines vertically positioned Canine cusp tips at the middle of roots the adjacent incisors (2.90±1.08). Females showed more difficulty regarding dental midline coincidence relative to the skeletal midline of the impacted as in most of the cases the dental midlines were shifted to one side of the dental arch (1.25±0.43). The same extend to the incisors irregularity or crowding of incisor segment (0.95±0.29).

## Discussion

Assessment Knowledge of dental anomalies in patients is mandatory for treatment planning (9). General dental practitioners who are aware of ethnic differences in the occurrence of dental anomalies are aware in finding them in patients during routine examinations, allowing for clinical intervention to avoid future complications (10,11). Canine impaction is one of the anomalies that should be considered by clinicians in details. The prevalence of maxillary canine impaction is variant among different populations (12-14). Different incidence was reported among different ethnic groups (15). Ethnic background of the population may result in lower or higher rates of some dental anomalies (16). There are limited studies (17,18) in the literature related to maxillary canine impaction in Saudi Arabia, but no study conducted in Jazan population as a different ethnic group among other ethnic groups in this country and no comprehensive study are available in the whole country to assess the severity of impaction and the orthodontic treatment difficulty.

The prevalence of maxillary canine impaction was 1.9% which is within the range of impacted canine in orthodontic literature (0.8 to 2.8%) (12-14). This percentage was close to that found in other studies in Saudi Arabia. El-Khateeb *et al.* (18) and Afify and Zawawi (17) found 1.6, 1.44 and 3.3% prevalence of impacted maxillary canines in Al-Madinah, Abha and Jeddah, respectively. Bilateral impaction was present in 22.3%, this is similar to the 25% and 19.2% observed in the Mexican (19) and Greek (1) population, respectively. The maxillary canines were ten time more prevalent than mandibular canine impactions. There is diversity of results in this aspect as some studies found five times (18) while other found seventeen times (17) differences in incidence of impaction of both impactions.

Canine impaction was twice as common in females (69.4%) as in males (30.6%); this finding is unlike to that found by others (18). Our result (2.26:1) is quite similar to most of the global studies in this context. The ratio of females to males is 2.3:1 in the group of American patients, 2.5:1 in Israeli patients (20) and 2.4:1 in Greek (1) population. Assessment of the severity of impaction irrelevant of the weight of each factor during orthodontic treatment revealed that there were no statistical significant differences between both genders in all evaluation factors.

The prognostic factors of impacted maxillary canines have been investigated by Pitt *et al.* (8) and McSherry (21) who suggested the use of these factors in an index to estimate orthodontic treatment difficulty. The result of the effect of the nine factors with their weight is listed in table 4. Patient age is an important factor for forced eruption is in childhood or adolescence because as the age increases, the impacted tooth may develops ankylosis and the chances of orthodontic traction become more difficult (22). This factor did not show any significant difference between both genders in this study.

Although canine impaction is more prevalent in females than males, the orthodontic treatment is significantly difficult in males regarding the angulation of impacted canines to the midsagittal plane, canine vertical position relative to the adjacent incisors and rotation of the impacted tooth. As angulation to the midline increases so does the likelihood of surgical removal rather than an attempt for forced eruption (23).

Regarding the vertical height of the canine crown, the more apical the position of the crown, the poorer the prognosis for orthodontic treatment. A good prognosis can be expected if the canine cusp tip is at the level of the cementoenamel junction of the adjacent incisor. A fair prognosis would be predicted for a canine with its cusp tip at a level of half the root length of the adjacent incisor, whilst a canine with poor prognosis for alignment would be one where the cusp tip lay against the apical third of the adjacent incisor root. It has been suggested that when the position of the canine tip is less than 14 mm above the occlusal plane, orthodontic treatment takes an average of 24 months; this increases to 31 months for vertical displacements beyond 14 mm (24).

Females showed more difficulty index in alignment of the incisors and maxillary midline shift, this is an indicated of more anterior crowding in females and the possibility of deciduous canine extraction and subsequent midline shifting. Both factors complicate the orthodontic treatment as both cases need more space for correction that sometimes necessitate therapeutic extraction. All and all the difficulty index for females is less than that in males.

## Conclusions

• Prevalence of maxillary canine impaction in Jazan is within the range of impacted canine in other populations.

• Canine impaction was twice as common in females as in males while the orthodontic treatment difficulty index is more in males than females.
